# Lack of Association between *ABO*, *PPAP2B*, *ADAMST7*, *PIK3CG*, and *EDNRA* and Carotid Intima-Media Thickness, Carotid Plaques, and Cardiovascular Disease in Patients with Rheumatoid Arthritis

**DOI:** 10.1155/2014/756279

**Published:** 2014-03-25

**Authors:** Raquel López-Mejías, Fernanda Genre, Mercedes García-Bermúdez, Begoña Ubilla, Santos Castañeda, Javier Llorca, Carlos González-Juanatey, Alfonso Corrales, José A. Miranda-Filloy, Trinitario Pina, Carmen Gómez-Vaquero, Luis Rodríguez-Rodríguez, Benjamín Fernández-Gutiérrez, Alejandro Balsa, Dora Pascual-Salcedo, Francisco J. López-Longo, Patricia Carreira, Ricardo Blanco, Javier Martín, Miguel A. González-Gay

**Affiliations:** ^1^Epidemiology, Genetics and Atherosclerosis Research Group on Systemic Inflammatory Diseases, Rheumatology Division, IDIVAL, Avenida Cardenal Herrera Oria s/n, 39011 Santander, Spain; ^2^Institute of Parasitología and Biomedicina López-Neyra, IPBLN-CSIC, Parque Tecnológico de Ciencias de la Salud, Avenida del Conocimiento s/n, Armilla, 18100 Granada, Spain; ^3^Department of Rheumatology, Hospital Universitario la Princesa, IIS-Princesa, Diego de León 62, 28006 Madrid, Spain; ^4^Department of Epidemiology and Computational Biology, School of Medicine, University of Cantabria, and CIBER Epidemiología y Salud Pública (CIBERESP), IDIVAL, Avenida Cardenal Herrera Oria s/n, 39011 Santander, Spain; ^5^Cardiology Division, Hospital Universitario Lucus Augusti, Doctor Ochoa s/n, 27004 Lugo, Spain; ^6^Department of Rheumatology, Hospital Universitario Lucus Augusti, Doctor Ochoa s/n, 27004 Lugo, Spain; ^7^Department of Rheumatology, Hospital Universitario Bellvitge, Feixa Llarga s/n, 08907 Barcelona, Spain; ^8^Department of Rheumatology, Hospital Clínico San Carlos, Profesor Martín Lagos s/n, 28040 Madrid, Spain; ^9^Department of Rheumatology, Hospital Universitario La Paz, Paseo de la Castellana 261, 28046 Madrid, Spain; ^10^Department of Rheumatology, Hospital General Universitario Gregorio Marañón, Doctor Esquerdo 46, 28007 Madrid, Spain; ^11^Department of Rheumatology, Hospital Universitario 12 de Octubre, Avenida de Córdoba s/n, 28041 Madrid, Spain

## Abstract

*Introduction*. Rheumatoid arthritis (RA) is a polygenic disease associated with accelerated atherosclerosis and increased cardiovascular (CV) mortality. Recent studies have identified the *ABO* rs579459, *PPAP2B* rs17114036, and *ADAMTS7* rs3825807 polymorphisms as genetic variants associated with coronary artery disease and the *PIK3CG* rs17398575 and *EDNRA* rs1878406 polymorphisms as the most significant signals related to the presence of carotid plaque in nonrheumatic Caucasian individuals. Accordingly, we evaluated the potential relationship between these 5 polymorphisms and subclinical atherosclerosis (assessed by carotid intima-media thickness (cIMT) and presence/absence of carotid plaques) and CV disease in RA. *Material and Methods*. 2140 Spanish RA patients were genotyped for the 5 polymorphisms by TaqMan assays. Subclinical atherosclerosis was evaluated in 620 of these patients by carotid ultrasonography technology. *Results*. No statistically significant differences were found when each polymorphism was assessed according to cIMT values and presence/absence of carotid plaques in RA, after adjusting the results for potential confounders. Moreover, no significant differences were obtained when RA patients were stratified according to the presence/absence of CV disease after adjusting for potential confounders. *Conclusion*. Our results do not confirm association between *ABO* rs579459, *PPAP2B* rs17114036, *ADAMTS7* rs3825807, *PIK3CG* rs17398575, and *EDNRA* rs1878406 and subclinical atherosclerosis and CV disease in RA.

## 1. Introduction

Rheumatoid arthritis (RA) is an autoimmune disease characterized by inflammation of synovial tissues in the joints and pannus formation and erosion [1]. This pathology affects up to 1% of the Caucasian population [2] and is associated with increased risk of cardiovascular (CV) disease and CV death [3]. This is the result of a process of accelerated atherosclerosis [4]. Although the etiology of RA is still unknown, traditional CV risk factors, chronic inflammation [5], and genetic background [6–9] have been implicated in the augmented CV mortality detected in this disorder. Subclinical atherosclerosis has been observed in patients with RA [10], even in those without traditional CV risk factors [10]. In this regard, both the assessment of carotid intima-media thickness (cIMT) and the presence of carotid plaques have been proposed to be useful markers as predictors of CV events in low and intermediate risk groups of nonrheumatic individuals [11] and in RA patients [12, 13].

Recently, a large-scale study of coronary artery disease (CAD) performed in nonrheumatic Caucasian individuals has identified 13 novel loci harboring one or more polymorphisms that were associated with this pathology. Moreover, this study confirmed the association of 10 out of 12 previously reported CAD loci [14]. With respect to this, the polymorphisms* ABO* (*histoblood group ABO system transferase*) rs579459,* PPAP2B* (*phosphatidic acid phosphatase type 2B*) rs17114036, and* ADAMTS7* (*metallopeptidase with thrombospondin type 1 motif, 7*) rs3825807 have been identified as significant genetic variants associated with CAD in nonrheumatic Caucasian individuals [14]. In addition, a recent meta-analysis of carotid intima-media thickness (cIMT) and presence/absence of carotid plaque performed in nonrheumatic Caucasian individuals has identified five new loci for common cIMT and plaque [15]. In this regard, the polymorphisms* PIK3CG* (*phosphatidylinositol-4,5-bisphosphate 3-kinase catalytic subunit gamma isoform*) rs17398575 and* EDNRA* (*endothelin receptor type A*) rs1878406 have been revealed as the most significant signals associated with presence of carotid plaques in nonrheumatic Caucasian individuals [15].

Taking all these considerations together, we aimed to determine, for the first time, the potential association between the* ABO* rs579459,* PPAP2B* rs17114036,* ADAMTS7* rs3825807,* PIK3CG* rs17398575, and* EDNRA* rs1878406 polymorphisms and subclinical atherosclerosis (assessed by the evaluation of cIMT values and presence/absence of carotid plaques) and CV disease in a large and well-characterized cohort of RA patients.

## 2. Patients and Methods

### 2.1. Patients and Study Protocol

For this purpose, a set of 2140 Spanish patients with RA were included in the present study. Blood samples were obtained from patients recruited from Hospital Lucus Augusti (Lugo), Hospital Marqués de Valdecilla (Santander), Hospital de Bellvitge (Barcelona), and Hospital Clínico San Carlos, Hospital La Paz, Hospital La Princesa, Hospital Gregorio Marañón and Hospital 12 de Octubre (Madrid). A subject's written consent was obtained in all the cases. The Ethics Committees of the corresponding hospitals approved the purpose of the work. All the patients fulfilled the 1987 American College of Rheumatology (ACR) and the 2010 classification criteria for RA [16, 17]. In all the cases, patients were assessed for* ABO* rs579459,* PPAP2B* rs17114036,* ADAMTS7* rs3825807,* PIK3CG* rs17398575 and* EDNRA *rs1878406 polymorphisms. In addition, cIMT and presence/absence of carotid plaques were determined by carotid ultrasonography (US) in 620 of these patients.

Information on the main demographic data, clinical characteristics, CV risk factors, and CV events of patients enrolled in the study is shown in Table 1. Three hundred and seventy-seven (17.6%) of these 2140 patients had experienced CV events. Definitions of CV events and traditional CV risk factors were established as previously described [4, 13].

### 2.2. Genotyping

DNA from patients was obtained from peripheral blood using standard methods.

The* ABO* rs579459,* PPAP2B* rs17114036,* ADAMTS7* rs3825807,* PIK3CG* rs17398575, and* EDNRA* rs1878406 polymorphisms were genotyped with TaqMan predesigned single-nucleotide polymorphism genotyping assays in a 7900 HT Real-Time polymerase chain reaction (PCR) system, according to the conditions recommended by the manufacturer (Applied Biosystems, Foster City, CA, USA). Negative controls and duplicate samples were included to check the accuracy of genotyping.

### 2.3. Carotid US Examination

Measurement of the cIMT and presence/absence of carotid plaques were performed in 620 patients from Lugo and Santander by carotid US. Patients from Santander were assessed using a commercially available scanner, MyLab 70, Esaote (Genoa, Italy), equipped with 7–12 MHz linear transducer and the automated software-guided technique radiofrequency—Quality Intima Media Thickness in real-time (QIMT, Esaote, Maastricht, Holland)—was used [18]. Patients from Lugo were assessed using high-resolution B-mode ultrasound, Hewlett Packard SONOS 5500, with a 10-MHz linear transducer as previously reported [19]. cIMT was measured at the far wall of the right and left common carotid arteries, 10 mm from the carotid bifurcation, over the proximal 15 mm long segment. cIMT was determined as the average of three measurements in each common carotid artery. The final cIMT was the largest average cIMT (left or right). The plaque criteria in the accessible extracranial carotid tree (common carotid artery, bulb, and internal carotid artery) were cIMT >1.5 mm, protrusion at least 50% greater than the surrounding cIMT, or arterial lumen encroaching >0.5 mm, according to Mannheim consensus criteria [20]. The carotid plaques were counted in each territory and defined as no plaque, unilateral plaque, or bilateral plaques [18, 20–22]. Agreement between the two US methods in patients with RA was previously reported [21]. Two experts with high experience and close collaboration in the assessment of subclinical atherosclerosis in RA from Santander (AC) and Lugo (CGJ) performed the studies.

### 2.4. Statistical Analysis

Statistical power for CV events was calculated using CaTS—Power Calculator for Two Stage Association Studies (http://www.sph.umich.edu/csg/abecasis/CaTS/). All genotype data were checked for deviation from Hardy-Weinberg equilibrium (HWE) using http://ihg.gsf.de/cgi-bin/hw/hwa1.pl.

cIMT values are displayed as mean and standard deviation (SD). The association between genotypes and alleles of each polymorphism and cIMT values was tested using unpaired *t*-test to compare between 2 groups and one-way analysis of variance (ANOVA) to compare more than two groups. Comparisons of means were adjusted for sex, age at the time of US study, follow-up time, and traditional CV risk factors (hypertension, diabetes mellitus, dyslipidemia, obesity, and smoking habit) as potential confounders using analysis of covariance (ANCOVA).

Differences in the genotypic and allelic frequencies of each polymorphism according to the presence/absence of carotid plaques were calculated by *χ* ^2^ or Fisher tests when necessary (expected values below 5). Strengths of associations were estimated using odds ratios (OR) and 95% confidence intervals (CI). Results were adjusted for sex, age at the time of US study, and traditional CV risk factors by logistic regression.

The relationship between genotypes and alleles of each polymorphism and CV events that occurred in the follow-up was tested using Cox regression adjusted for sex, age at RA diagnosis, and traditional CV risk factors. For that purpose, we used the most frequent genotype and allele as reference; the end of follow-up was the first date among the end of the study, date of death, or date of CV event. Follow-up time was estimated as the difference between the RA diagnosis date and the end of follow-up. Patients without CV events in the follow-up time or dying by any non-CV cause were considered as censored. Results were expressed as hazard ratios (HR) with 95% confidence interval (CI).

Statistical significance was defined as *P* < 0.05. All analyses were performed with STATA statistical software 12/SE (Stata Corp., College Station, TX, USA).

## 3. Results 

This study had >80% of power to detect genotypic OR >1.4 for* ABO* rs579459,* PPAP2B* rs17114036,* ADAMTS7* rs3825807,* PIK3CG* rs17398575, and* EDNRA* rs1878406.

The* ABO* rs579459,* PPAP2B* rs17114036,* ADAMTS7* rs3825807,* PIK3CG* rs17398575, and* EDNRA* rs1878406 genotype distributions were in Hardy-Weinberg equilibrium. The genotyping success was greater than 97% in all the cases.

Table 2 describes the potential association between the* ABO* rs579459,* PPAP2B* rs17114036,* ADAMTS7* rs3825807,* PIK3CG* rs17398575, and* EDNRA* rs1878406 polymorphisms and both subclinical atherosclerosis and CV disease in RA patients. As shown in Table 2, no statistically significant differences were found when each polymorphism was assessed according to the evaluation of the cIMT in RA patients, after adjusting the results for sex, age at the time of US study, and traditional CV risk factors (hypertension, diabetes mellitus, dyslipidemia, obesity, and smoking habit) as potential confounders. Similarly, no statistically significant differences were detected when each polymorphism was evaluated according to the presence/absence of carotid plaques in RA, after adjusting the results for potential cofounder factors (Table 2). Moreover, when RA patients were stratified according to the presence or absence of CV disease, no significant differences were obtained after adjusting the results for sex, age at RA diagnosis, and traditional CV risk factors (Table 2). It was also the case when RA patients were stratified according to the presence/absence of ischemic heart disease (data not shown).

## 4. Discussion 

CV disease is the most common cause of premature mortality in patients with RA being a consequence of accelerated atherosclerosis [23]. Since a genetic background [6–9] has been involved in the accelerated atherosclerosis in RA, several studies have been focused on the search of genetic markers that may improve the identification of RA patients at risk of experiencing CV events.

Previous studies have described the* ABO* rs579459,* PPAP2B* rs17114036, and* ADAMTS7* rs3825807 polymorphisms as genetic variants associated with coronary artery disease [14] and the* PIK3CG* rs17398575 and* EDNRA* rs1878406 polymorphisms as the most significant signals related to the presence of carotid plaque [15] in nonrheumatic Caucasian individuals. Taking into account these considerations, we assessed for the first time the potential association between these 5 polymorphisms and subclinical atherosclerosis and CV events in RA patients.

Nevertheless, in contrast to the results obtained in nonrheumatic Caucasian individuals [14], the relationship between the* ABO* rs579459,* PPAP2B *rs17114036,* ADAMTS7* rs3825807,* PIK3CG* rs17398575, and* EDNRA* rs1878406 polymorphisms and CV events was not statistically significant. Moreover, unlike nonrheumatic individuals [15], we did not disclose association between the* ABO rs579459, PPAP2B rs17114036, ADAMTS7 rs3825807, PIK3CG rs17398575*, and* EDNRA rs1878406 *polymorphisms and cIMT values or presence/absence of carotid plaques.

Although atherosclerosis and RA are chronic inflammatory diseases that share similar pathophysiological mechanisms [24], additional factors so far unknown may explain the differences observed in terms of genetic predisposition. Further studies to better characterize the genetic susceptibility for accelerated atherosclerosis in RA are under way.

## 5. Conclusion

Our results do not confirm association of* ABO* rs579459,* PPAP2B* rs17114036,* ADAMTS7* rs3825807,* PIK3CG* rs17398575, and* EDNRA* rs1878406 polymorphisms with subclinical atherosclerosis and CV disease in RA patients.

## Figures and Tables

**Table 1:** Demographic and clinical characteristics of the Spanish patients with RA included in the study.

Clinical feature	% (*n*/*N*)
Patients	2140
Main characteristics	
Age at the time of disease onset (years, mean ± SD)	52.4 ± 14.9
Follow-up (years, mean ± SD)	12.2 ± 8.8
Percentage of women	75.1
Rheumatoid factor positive*	67.9 (1388/2044)
Anti-CCP antibodies positive	59.2 (1058/1786)
Shared epitope positive	62.4 (736/1180)
Erosions	54.7 (940/1718)
Extra-articular manifestations**	31.5 (540/1715)
Cardiovascular risk factors	
Hypertension	38.7 (817/2108)
Diabetes mellitus	12.2 (258/2108)
Dyslipidemia	37.3 (786/2108)
Obesity	16.8 (355/2108)
Smoking habit	23.8 (502/2108)
Patients with cardiovascular events	17.6 (377/2140)
Ischemic heart disease	8.3 (179/2140)
Heart failure	5.3 (113/2140)
Cerebrovascular accident	5.3 (114/2140)
Peripheral arteriopathy	2.3 (50/2140)

RA: rheumatoid arthritis; SD: standard deviation; Anti-CCP antibodies: anticyclic citrullinated peptide antibodies.

*At least two determinations were required for analysis of this result.

**Extra-articular manifestations of the disease (if RA patients experienced at least one of the following manifestations: nodular disease, Felty's syndrome, pulmonary fibrosis, rheumatoid vasculitis, or secondary Sjögren's syndrome) [4].

**Table 2:** Association between *AB0, PPAP2B, ADAMTS7, PIK3CG*, and *EDNRA* genotypes and alleles and cIMT, presence/absence of carotid plaques, and CV events in RA patients.

	cIMT mm (*n* = 620)		Presence/absence of carotid plaques (*n* = 620)		Presence/absence of CV events (*n* = 2140)	
	Mean ± SD	P*	P**	OR [95% CI]**	P***	HR [95% CI]***
*ABO* rs579459						
TT	0.73 ± 0.17	0.91	—	1 (Ref.)	—	1 (Ref.)
TC	0.74 ± 0.17		0.23	0.66 [0.33–1.30]	0.79	1.05 [0.72–1.53]
CC	0.71 ± 0.17		0.63	0.71 [0.18–2.86]	0.22	0.59 [0.26–1.37]
T	0.74 ± 0.17	0.86	—	1 (Ref.)	—	1 (Ref.)
C	0.73 ± 0.17		0.29	0.76 [0.45–1.26]	0.47	0.89 [0.67–1.20]
*PPAP2B* rs17114036						
AA	0.74 ± 0.17	0.64	—	1 (Ref.)	—	1 (Ref.)
AG	0.73 ± 0.19		0.55	1.14 [0.71–1.86]	0.99	0.99 [0.58–1.70]
GG	0.74 ± 0.23		0.10	0.72 [0.03–1.54]	*¥*	*¥*
A	0.74 ± 0.17	0.99	—	1 (Ref.)	—	1 (Ref.)
G	0.74 ± 0.19		0.10	0.49 [0.21–1.15]	0.48	0.83 [0.50–1.39]
*ADAMTS7 *rs3825807						
AA	0.76 ± 0.17	0.46	—	1 (Ref.)	—	1 (Ref.)
AG	0.73 ± 0.17		0.44	0.86 [0.58–1.28]	0.10	0.59 [0.39–1.20]
GG	0.72 ± 0.17		0.72	0.92 [0.57–1.49]	0.73	0.92 [0.56–1.49]
A	0.74 ± 0.17	0.34	—	1 (Ref.)	—	1 (Ref.)
G	0.73 ± 0.17		0.53	0.86 [0.55–1.36]	0.43	0.89 [0.69–1.17]
*PIK3CG *rs17398575						
GG	0.73 ± 0.16	0.1	—	1 (Ref.)	—	1 (Ref.)
GA	0.74 ± 0.18		0.34	1.38 [0.72–2.64]	0.99	1.00 [0.68–1.47]
AA	0.75 ± 0.19		0.86	0.86 [0.17–4.26]	0.95	1.02 [0.47–2.23]
G	0.73 ± 0.17	0.09	—	1 (Ref.)	—	1 (Ref.)
A	0.74 ± 0.18		0.56	1.17 [0.70–1.95]	0.96	1.00 [0.75–1.36]
*EDNRA* rs1878406						
CC	0.73 ± 0.16	0.61	—	1 (Ref.)	—	1 (Ref.)
CT	0.74 ± 0.19		0.28	1.54 [0.70–3.38]	0.44	0.83 [0.53–1.32]
TT	0.73 ± 0.14		0.80	0.76 [0.10–6.04]	0.85	1.10 [0.40–2.90]
C	0.74 ± 0.16	0.32	—	1 (Ref.)	—	1 (Ref.)
T	0.74 ± 0.18		0.47	1.28 [0.66–2.47]	0.62	0.91 [0.62–1.33]

cIMT: carotid intima-media thickness; CV: cardiovascular; RA: rheumatoid arthritis; SD: standard deviation; OR: odds ratio; CI: confidence interval; HR: hazard ratios.

*Adjusted for sex, age at the time of ultrasonography study, follow-up time, and traditional CV risk factors (hypertension, diabetes mellitus, dyslipidemia, obesity, and smoking habit) using analysis of covariance (ANCOVA).

**Adjusted for sex, age at the time of ultrasonography study, follow-up time, and traditional CV risk factors (hypertension, diabetes mellitus, dyslipidemia, obesity, and smoking habit) by logistic regression.

***Adjusted for sex, age at RA diagnosis, follow-up time, and traditional CV risk factors (hypertension, diabetes mellitus, dyslipidemia, obesity, and smoking habit) using Cox regression.

^*¥*^Model does not converge.
